# Whole-Genome Analysis of the Influenza A(H1N1)pdm09 Viruses Isolated from Influenza-like Illness Outpatients in Myanmar and Community-Acquired Oseltamivir-Resistant Strains Present from 2015 to 2019

**DOI:** 10.3390/v16081300

**Published:** 2024-08-15

**Authors:** Irina Chon, Su Mon Kyaw Win, Wint Wint Phyu, Reiko Saito, Yadanar Kyaw, Nay Chi Win, Di Ja Lasham, Htay Htay Tin, Tsutomu Tamura, Teruhime Otoguro, Keita Wagatsuma, Yuyang Sun, Jiaming Li, Hisami Watanabe

**Affiliations:** 1 Division of International Health (Public Health), Graduate School of Medical and Dental Sciences, Niigata University, Niigata 951-8510, Japan; jasmine@med.niigata-u.ac.jp (R.S.); waga@med.niigata-u.ac.jp (K.W.); sunyuyang@med.niigata-u.ac.jp (Y.S.); lijiaming@med.niigata-u.ac.jp (J.L.); 2 Infectious Diseases Research Center of Niigata University in Myanmar (IDRC), Graduate School of Medical and Dental Sciences, Niigata University, Niigata 951-8510, Japan; sumonkyawwin@gmail.com (S.M.K.W.); dr.naychiwin@gmail.com (N.C.W.); lashamdidi2010@gmail.com (D.J.L.); ttamura@med.niigata-u.ac.jp (T.T.); totoguro@med.niigata-u.ac.jp (T.O.); hwatanabe@med.niigata-u.ac.jp (H.W.); 3 Department of Microbiology, University of Medicine, Magway 04012, Myanmar; wintwintphyu2012@gmail.com; 4 Respiratory Medicine Department, Thingangyun Sanpya General Hospital, Yangon 110-71, Myanmar; ydkkyaw@gmail.com; 5 University of Medical Technology, Yangon 110-12, Myanmar; drhtayhtaytin@gmail.com; 6 Institute for Research Administration, Niigata University, Niigata 951-8510, Japan

**Keywords:** influenza A(H1N1)pdm09, next-generation sequencing, whole-genome analysis, genetic reassortment, neuraminidase inhibitors, antiviral resistance, H275Y

## Abstract

In this study, we describe the genetic characteristics of influenza A(H1N1)pdm09 strains detected in Myanmar from 2015 to 2019. Whole genomes from 60 A(H1N1)pdm09 virus isolates were amplified using real-time polymerase chain reaction and successfully sequenced using the Illumina iSeq100 platforms. Eight individual phylogenetic trees were retrieved for each segment along with those of the World Health Organization (WHO)-recommended Southern Hemisphere vaccine strains for the respective years. A(H1N1)pdm09 viruses from 2015 were found to belong to clade 6B, those from 2016 to 6B.1, 2017 to 6B.1A, and 2019 to 6B.1A.5a, and were genetically distinct from the Southern Hemisphere vaccine strains for the respective seasons, A/California/7/2009 and A/Michigan/45/2015. We observed one virus with intra-subtype reassortment, collected in the 2015 season. Importantly, three viruses possessed the H275Y substitution in the neuraminidase protein, appearing to be community-acquired without the prior administration of neuraminidase inhibitors. These viruses exhibited highly reduced susceptibility to oseltamivir and peramivir. This study demonstrates the importance of monitoring genetic variations in influenza viruses that will contribute to the selection of global influenza vaccines.

## 1. Introduction

Influenza viruses cause an estimated 3 to 5 million cases of severe respiratory illness each year, resulting in approximately 290,000 to 650,000 deaths annually [1]. In developing countries, nearly 99% of children under 5 years old die from influenza-related lower respiratory infections [2].

The genome of the influenza virus consists of eight gene segments: the RNA polymerase subunits polymerase basic 2 (PB2), polymerase subunits polymerase basic 1 (PB1), and polymerase acidic (PA), which encode the viral RNA-dependent RNA polymerase as well as proteins implicated in inducing cell death (e.g., PB1- F2) and modulate viral pathogenicity (e.g., PA-X^9^). Additionally, the genome encodes hemagglutinin (HA); nucleoprotein (NP); neuraminidase (NA); matrix proteins (M); and non-structural proteins (NS) [3]. Among the four types of influenza viruses: A, B, C, and D [4], influenza A and B viruses circulate and cause seasonal epidemics in humans [5]. Influenza A viruses are further classified based on the surface glycoproteins HA and NA. The HA protein mediates virus attachment and entry into the host cell, while NA protein facilitates virus release after budding, thereby contributing to the spread of infection [4].

In April 2009, a new swine-origin influenza virus A(H1N1)pdm09 emerged in Mexico and the United States, which caused a rapid global spread by human-to-human transmission, leading the World Health Organization (WHO) to declare a phase 6 pandemic alert on 11 June 2009 [6]. A(H1N1)pdm09 emerged through a novel reassortment event involving North American and Eurasian swine influenza viruses. It contains PB2, PB1, PA, HA, NP, and NS segments from North American triple-reassortment swine viruses, and NA and M segments derived from the Eurasian swine lineage [7]. Meanwhile, Southeast Asia is considered an epicenter for influenza pandemics throughout history [8]. The region’s unique environment and lifestyle foster close contact between wild aquatic birds, domestic poultry, pigs, and humans, creating opportunities for interspecies transmission and the generation of new reassortment influenza viruses [9].

The influenza seasonality pattern in Myanmar typically starts in May or June, peaks during July or August, and finishes between September and October [10]. The WHO designated the National Influenza Centre (NIC) in the National Health Laboratory (NHL), Yangon, Myanmar, on 26 January 2008. Initially, two influenza surveillance sites in Myanmar, one in Yangon and another in Pyinmana, Nay Pyi Taw, were established. With the support of the WHO, the NIC of Myanmar strengthened the national influenza surveillance system together with the Central Epidemiology Unit (CEU) under the Department of Public Health, Ministry of Health, in 2018. The NIC of Myanmar allocated new influenza surveillance sites in eight state and region hospitals. At least five samples of influenza-like illness (ILI) and all cases of severe acute respiratory infections are sent from each surveillance site to the NIC, Myanmar, on a weekly basis to assess the influenza circulation in the country [11]. Influenza vaccination is rare in Myanmar since there is no government-supplied vaccination program for the influenza virus. The influenza vaccine can be administered in private clinics as a travel vaccination for those visiting colder-climate countries.

Our group has been working on human influenza virus surveillance in Myanmar for nearly two decades [10,12,13,14,15,16,17,18,19,20]. During the 2010–2015 seasons, we reported activities of influenza A(H1N1)pdm09, A(H3N2), and B in Myanmar [10]. The Myanmar A(H1N1)pdm09 viruses then evolved from clade 4 to 6B, with no substitutions found that reduce inhibition to neuraminidase inhibitors (NAIs) [10]. In 2017, between July and August, Myanmar experienced a larger outbreak, with severe pneumonia caused by A(H1N1)pdm09. Strains collected during the 2017 outbreaks, which underwent next-generation sequencing (NGS), belonged to the 6B.1 clade [16]. The first oseltamivir-resistant A(H1N1)pdm09 virus with an H275Y variant in NA was found from a case with no prior history of oseltamivir administration during the outbreak in 2017 [16]. Usually in Myanmar, anti-influenza agents are not prescribed as the treatment for influenza infection. However, in 2017, during an outbreak, the WHO supplied oseltamivir as an emergency control measure for patients with severe cases. Unfortunately, the number of prescribed cases is not available.

NGS technologies are employed for the molecular surveillance of influenza viruses in many countries [21,22,23]. As the role of intra-subtype reassortment becomes increasingly evident, identifying reassortments with whole-genome-based surveillance is the gold standard, particularly for recombinogenic viruses like influenza. However, comprehensive data on the whole genome of the influenza virus are generally lacking in Myanmar.

We previously reported influenza A(H3N2) evolution dynamics during the 2015–2019 seasons in Myanmar by performing a whole genetic analysis of eight segments [17]. This study aims to compare the whole-genome sequences of influenza A(H1N1)pdm09 viruses circulating in Myanmar from 2015 to 2019. We compared circulating A(H1N1)pdm09 strains in Myanmar with the WHO-recommended Southern Hemisphere (SH) vaccine strains of the respective years to detect the emergence of new influenza virus variants. In addition, we screened the NA-H275Y variant in A(H1N1)pdm09 viruses that confer resistance to oseltamivir and peramivir, which cause potential human-to-human transmission in Myanmar, where anti-influenza drugs are rarely used. Our data will contribute to global surveillance for selecting influenza vaccine strains and detecting new variants, including reassortant or antiviral drug-resistant viruses.

## 2. Materials and Methods

### 2.1. Study Population

Outpatients presenting with ILI symptoms, who visited two surveillance site hospitals (Thinganguyn General Hospital in Yangon and the 200-bed General Hospital in Pyinmana, Myanmar), between January 2015 and December 2019, were enrolled in the current study. The inclusion criteria for symptoms of ILI patients comprised the sudden onset of fever (>37.8 °C) and at least two of the following symptoms: cough, rhinorrhea, myalgia, arthralgia, and diarrhea. The exclusion criteria included suspected cases of digestive tract infections and chronic respiratory infections such as tuberculosis. Written informed consents were obtained from all the patients or their guardians who agreed to participate in the study. Personal information such as name, age, sex, address, and clinical details including the date of symptom onset, date of clinic visit, influenza vaccination history, anti-viral medication history, and clinical symptoms, were recorded in clinical data sheets prior to sample collection.

### 2.2. Sample Collection

Two nasopharyngeal swabs were collected from each ILI patient who participated in the study. One sample was screened for influenza virus using the Quick-Navi Flu+RSV rapid diagnostic test (Denka Seiken, Tokyo, Japan). If the rapid diagnostic test was positive for influenza A or B, the other sample was placed in a viral transport medium and frozen at −20 °C at the study sites. All samples collected in viral transport media were transferred and stored at −80 °C at the NHL in Yangon until international transportation. Subsequently, the samples were transported to the Division of International Health (Public Health), Graduate School of Medical and Dental Sciences, Niigata University, Niigata, Japan, for further laboratory examination. This study received ethical approval from the Niigata University Ethical Committee (2015–2533) and the Ethical Review Committee of the Department of Medical Research, Ministry of Health and Sports, Myanmar (No. 016516).

### 2.3. Virus Isolation and Subtype Identification

Influenza viruses were isolated by inoculating 100 μL of clinical samples into confluent Madin–Darby canine kidney (MDCK) cells during 2015 and 2017 and MDCK-SIAT1 cells in 2018 and 2019, and propagated in 48-well plates [17]. Since 2018, it has become globally challenging to isolate influenza A(H3N2) from MDCK cells [24,25]. Consequently, we transitioned to using MDCK-SIAT1 cells for influenza virus isolation in general, as they facilitate the easier proliferation of A(H3N2).

The confluent cells were inoculated with clinical specimens and incubated at 34 °C with 5% CO_2_. Cells were observed daily for 3–10 days to detect specific cytopathic effects (CPE) [26]. If no CPEs were observed during the initial inoculation, samples underwent a second passage using the same cells. The culture supernatants and cells that showed CPEs were collected and stored at −80 °C. Viral RNA was extracted from 140 μL of isolates using the QIAamp Viral RNA Mini Kit (QIAGEN, Hilden, Germany), followed by reverse transcription into complementary DNA (cDNA) as previously reported [27]. The obtained cDNA was then subjected to the cycling probe real-time PCR (RT-PCR), a method exclusively developed in our department to screen for the H275Y substitution in NA in A(H1N1)pdm09. Two fluorescent-labeled chimeric RNA-DNA probes with 6-carboxyfluorescein (FAM) and 6-carboxy-Xrhodamine (ROX) were designed to detect single-nucleic acid polymorphisms (SNPs), H275 or H275Y, in the NA gene of influenza A(H1N1)pdm09 [28].

### 2.4. Next-Generation Sequencing from the A(H1N1)pdm09 Isolates

For genetic characterization of the whole-genome sequences of influenza, 60 A(H1N1)pdm09 viruses were selected from 315 viruses recovered during the study period. Selection criteria for the virus isolates were those with a cycle threshold (C_t_) value ≤ 32 in PCR amplification and sampled in different months and dates. We did not consider the geographical representativeness of the isolates, whether they were collected from Yangon or Pyinmana.

Viral nucleic acids were extracted from the viral isolates using a High Pure Viral RNA Kit (Roche Diagnostics, Mannheim, Germany) following the manufacturer’s instructions. Sequencing amplicons were produced using one-step reverse transcription PCR (PrimeScript II High Fidelity, TaKaRa Bio, Shiga, Japan). Amplified products were purified and quantified before library preparation as previously reported [12,17]. Sequencing libraries were created and run on an Illumina platform (iSeq 100 Illumina, San Diego, CA, USA). Raw sequences (paired-end) approximately 250 bp in length were trimmed and assembled, using bioinformatics software (CLC Genomics Workbench bio software v.20.0.2, CLC bio, Cambridge, MA, USA) [12]. This approach allowed researchers to obtain nucleotide sequences for all eight viral segments, facilitating genetic analysis and the characterization of the influenza virus.

### 2.5. Subclade Classification by Amino Acid Substitutions in HA

The subclades of the HA gene from A(H1N1)pdm09 in this study were determined by the key amino acid substitutions, as proposed by the WHO Worldwide Influenza Centre report from the Crick Institute (https://www.crick.ac.uk/research/platforms-and-facilities/worldwide-influenza-centre/annual-and-interim-reports; accessed on 10 October 2023). The classification of subgroups under the 6B group, such as 6B.1, 6B.1A, and 6B.1A.5a, was determined by key amino acid substitutions based on A/California/7/2009, an SH vaccine strain used between 2015 and 2016, and A/Michigan/45/2015, an SH vaccine strain used during 2017 and 2019. The genetic analysis of the amino acid substitutions was performed using the Molecular Evolutionary Genetics Analysis (MEGA) software v.6.06 (https://www.megasoftware.net/; accessed on 10 October 2023). MEGA software was utilized to construct maximum-likelihood trees of the eight individual segments. The model applied was the TN93+G (Tamura’s three-parameter distance model with the gamma distribution), with 1000 bootstrap replicates.

The maximum-likelihood phylogenetic reconstructions for seven segments, PB2, PB1, PA, NP, NA, MP, and NS, were generated and the positions of subclades were compared among eight segments. Subclade classification for the seven segments other than HA was conducted based on the HA topology, using the SH vaccine strains as reference strains for their respective subclades. To detect reassortment, we compared the topologies of all trees generated by the eight segments against the phylogeny of the HA segment, identifying inconsistencies indicative of intra-subgroup reassortment.

We conducted a BLAST search using the Global Initiative on Sharing All Influenza Data (GISAID) for the HA gene of single A(H1N1)pdm09 sequences from each season to determine the genetic relationship between the Myanmar strains and globally circulating strains. Additionally, we constructed a phylogenetic tree of 210 A(H1N1)pdm09 strains using the GTR+G model with 1000 bootstrap replicates to visualize the clade classification of the HA segment and group clustering of the Myanmar and global viruses.

### 2.6. Vaccine Strains

The WHO-recommended SH vaccine strains from 2015 to 2019 were used as reference strains, and downloaded from the influenza research database (https://www.ncbi.nlm.nih.gov/genomes/FLU/Database/nph-select.cgi?go=databas; accessed on 12 June 2024). Whole genomes of the viruses circulating each year in Myanmar were aligned against the SH vaccine strain for the respective year. Viruses collected in 2015 and 2016 were mapped to A/California/7/2009 (EPI_ISL_391380), while those from 2017 to 2019 were mapped to A/Michigan/45/2015 (EPI_ISL_208220) by CLC Genomics Workbench. Amino acids obtained from the sequenced genomes of the eight virus genome segments (HA, NA, PB2, PB1, PA, NP, MP, and NS) were compared with each year’s vaccine strain using the MEGA software, to identify any differences between the vaccine strain and circulating strains in Myanmar.

### 2.7. NA Inhibitors Susceptibility Assay

The isolates of A(H1N1)pdm09 virus that were positive for H275Y in NA by the cycling probe real-time PCR assay were tested by fluorescence-based inhibition assay (NA inhibition assay) in order to measure the susceptibility to four kinds of NA inhibitors, peramivir (Shionogi & Co., Ltd., Osaka, Japan), oseltamivir (Sequoia Research Products Ltd., Pangbourne, UK), zanamivir (Sequoia Research Products Ltd.), and laninamivir (Daiichi Sankyo Co., Ltd., Tokyo, Japan). In addition, the selected number of isolates that were negative for H275Y substitution, as tested by the cycling probe real-time PCR assay, were tested with susceptibility assays for NAIs. Firstly, NA assay was carried out to adjust the optimal concentration of NA activity from the virus isolates. Then, the subsequent NA inhibition assay using the MUNANA (4-(methylumbelliferyl)-N-acetylneuraminic acid) fluorescence-based methods was conducted to determine the 50% inhibitory concentrations (IC_50_), as previously reported [16]. The strain was assessed as reduced inhibition (RI) if the IC_50_ value of one of the four drugs showed a 10- to 100-fold rise against the median values of all A(H1N1)pdm09 viruses tested, and highly reduced inhibition (HRI) if IC_50_ showed a >100-fold elevation [29]. In addition, substitutions in NA that confer reduced inhibition to NAIs, as reported by the WHO’s Antiviral Group, were screened from the genetic sequences obtained through NGS [30].

## 3. Results

### 3.1. Characterization of Influenza Virus Isolates

A total of 2058 nasal swabs were collected from outpatients with ILI in Myanmar from 2015 to 2019. Among the 1303 samples that tested positive in rapid diagnostic tests, 803 (61.6%) virus isolates were successfully recovered from MDCK or MDCK-SIAT1 cells. Of these 803 isolates, 444 (55.3%) were classified as influenza A, with 129 (29.1%) identified as A(H3N2) and 315 (70.9%) as A(H1N1)pdm09. The remaining 359 (44.7%) isolates were influenza B.

Sixty out of the 315 influenza A(H1N1)pdm09 virus isolates underwent NGS molecular genetic analysis, of which eighteen viruses were collected in 2015, six in 2016, twenty-one in 2018 and fourteen in 2019. Consensus genomes for all 60 A(H1N1)pdm09 isolates were successfully sequenced and those with >95% coverage were analyzed. The average mean depth ranged from 1000 to 3000 across all segments, although coverage generally declined with increasing segment amplicon size.

### 3.2. Subclades Analysis by Amino Acid Substitutions and Phylogenetic Tree Analysis of HA

**Figure 1:**
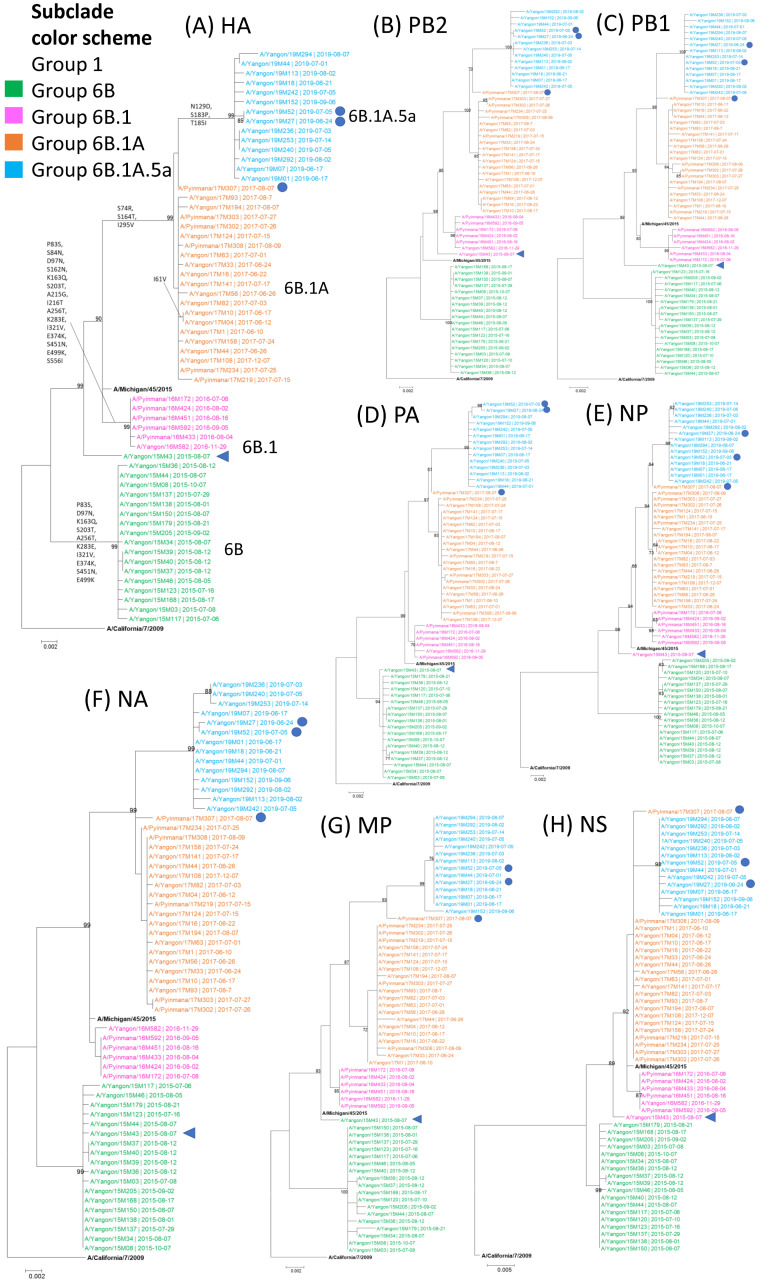
Maximum-likelihood phylogenetic trees of all eight segments of influenza A(H1N1)pdm09 viruses circulating in Myanmar from 2015 to 2019. The genetic sequences from 60 A(H1N1)pdm09 strains isolated in Myanmar between 2015 and 2019 were classified into subclades defined by the WHO, and compared to the Southern Hemisphere vaccine strains. The strains were colored by subclade, and the Southern Hemisphere vaccine strains during the study period are indicated in bold: (**A**) HA, (**B**) PB2, (**C**) PB1, (**D**) PA, (**E**) NP, (**F**) NA, (**G**) MP, and (**H**) NS segments. Amino acid substitutions, as compared to A/California/7/2009, were indicated at the nodes in the HA phylogeny. One strain, supposed to have an intra-subtype reassortment, A/Yangon/15M043/2015, is marked by a blue triangle. Three strains harboring the H275Y substitution, A/Pyinmana/17M307/2017, A/Yangon/19M027/2019, and A/Yangon/19M052/2019, are marked by a blue closed circle. The phylogenetic tree was inferred by the maximum-likelihood method using 1000 bootstrap replicates implemented by MEGA software v.7.0.26. The bootstrap values > 70% are indicated at the nodes.

The analysis of the phylogenetic tree revealed four distinct subclades (6B, 6B.1, 6B.1A, and 6B.1A.5a) within the A(H1N1)pdm09 subtype in Myanmar from 2015 to 2019 (Figure 1A), according to the key amino acid substitutions in HA defining subclades proposed by the WHO (https://www.crick.ac.uk/research/platforms-and-facilities/worldwide-influenza-centre/annual-and-interim-reports; accessed 12 October 2023). Compared to the vaccine strain, A/California/7/2009, the D97N, K163Q, S203T, A256T, and K283E amino acid substitutions in the HA of the Myanmar strains indicated that all strains in 2015 belonged to clade 6B (n = 20, 33%). In addition to substitutions defining the 6B clade, the S84N, S162N, and I216T substitutions were detected in all strains collected in 2016 and formed subclade 6B.1 (n = 6, 10%). Starting from 2017, aligning with A/Michigan/45/2015, all strains harbored the S74R, S164T, and I295V substitutions and belonged to subclade 6B.1A (n = 20, 33%). In 2019, several substitutions, including N129D and T185I, defined the Myanmar strains as belonging to subclade 6B.1A.5 (n = 14, 24%).

From 2015 to 2019, the HA segment in Myanmar was most closely related to the strains isolated in Thailand, Australia, China, Singapore, the USA, European countries, Laos, and Bangladesh, as determined by BLAST search (Figure S1). We noted that Myanmar’s A(H1N1)pdm09 viruses from 2015 and 2016, belonging to clades 6B and 6B.1, respectively, were closely related to strains from Southeast Asia, Oceania, and the USA. Subsequently, the viruses isolated from Myanmar in 2017 and 2019 were similar to those in Southeast Asia, China, and Europe, clustering in two clades: 6B.1A and 6B.1A.5a (Figure S1).

### 3.3. Comparison of Amino Acid Substitutions between Circulating Strains and Vaccine Strains in HA Segments

We compared amino acid substitutions of circulated A(H1N1)pdm09 viruses isolated from 2015 to 2016 with a SH vaccine strain—A/California/7/2009—and viruses from 2017 to 2019 with a SH vaccine strain—A/Michigan/45/2015.

In the HA segment of the circulating A(H1N1)pdm09 viruses isolated from 2015 and the SH vaccine strains of the relevant year, a total of 11 common amino acid substitutions (P83S, D97N, K163Q, S185T, S203T, A256T, K283E, I321V, E374K, S451N, and E499K) were observed. Six sporadic substitutions (S84N, S143G, N370S, T391I, K454R, and S556I) were additionally detected (Table 1). Among the 2016 isolates, only 16 common amino acid substitutions (P83S, S84N, D97N, S162N, K163Q, S185T, S203T, A215G, I216T, A256T, K283E, I321V, E374K, S451N, E499K, and S556I) were observed (Table 1).

The HA segment of the 2017 isolates in Myanmar were compared with the 2017 vaccine strain A/Michigan/45/2015. A total of three common amino acid substitutions (S74R, S164T, and I295V), and an additional five sporadic substitutions (D35G, I61V, G155E, A261V, and V449I) were detected in the HA segment of some of the 2017 isolates in Myanmar (Table 1). Similarly, the 2019 Myanmar isolates were compared with the 2019 SH vaccine strain of A/Michigan/45/2015. A total of six common amino acid substitutions (S74R, N129D, S164T, S183P, T185I, and I295V) were detected in all Myanmar isolates in 2019. Four additional sporadic substitutions (K146R, S478N, D501Y, and V528I) were also found in some of the 2019 isolates (Table 1). In general, the number of amino acid substitutions were the highest in 2015 and 2016, suggesting that these viruses mismatched with the vaccine strain for those seasons. However, we did not perform serological antigenic analyses, such as hemagglutination inhibition assays or neutralization tests, to confirm the antigenicity mismatches.

### 3.4. Analysis of Whole-Genome Sequence Data in Seven Segments

The maximum-likelihood phylogenetic tree reconstruction was built for seven segments, PB2, PB1, PA, NP, NA, MP, and NS, to demonstrate the topology of those segments in the circulated strains compared to the clade classification based on the HA segment (Figure 1B–H). Basically, the clustering of the strains in the seven segments on the phylogenetic tree was consistent with that of the HA segment: clade 6B, 6B.1, 6B.1A, and 6B.1A.5A. It was suggested that no group reassortment events occurred among the clades. However, one virus, A/Yangon/15M143, belonged to the 6B clade in the HA phylogenetic tree but showed a topology consistent with the 6B.1 clade in the PB2, NP, and NS segments (Figure 1B,E,H). These findings suggest that the virus demonstrated an intra-subtype reassortment event.

The analysis of amino acid mutation differences in seven segments compared with vaccine strains of respective years is presented in the Supplementary Tables (Tables S1–S7).

### 3.5. Community-Aquired Oseltamivir-Resistant Influenza A(H1N1)pdm09 Virus

Out of the 60 influenza A(H1N1)pdm09 viruses studied, three isolates (5.0%), A/Pyinmana/17M307/2017, A/Yangon/19M027/2019, and A/Yangon/19M052/2019, were found to have oseltamivir resistance due to the H275Y substitution in the NA segment. This H275Y substitution was initially detected by cycling probe real-time PCR assay and subsequently confirmed by NGS. In the remaining 57 viruses, NGS analysis confirmed that there were no substitutions in the NA segment conferring reduced susceptibility to NAIs.

Among the three patients harboring the NA/H275Y substitution, one was 28 years old, another was 5 years old, and the third was 5 months old. At their presentation to the study hospitals, their body temperatures were recorded at 37.0 °C, 37.2 °C, and 37.5 °C, respectively, with symptoms including cough, rhinorrhea, and headache. None of these patients had received influenza vaccination, and detailed information regarding their comorbidities was not available. Furthermore, none of the patients had undergone anti-influenza treatment prior to sample collection, suggesting a probable community-acquired infection with the NA/H275Y viruses. Information on recent exposure to other influenza patients treated with oseltamivir was also lacking. The patients did not receive oseltamivir treatment at the study hospitals, in line with the standard practice in Myanmar, where oseltamivir is not commonly administered unless patients are hospitalized.

The drug susceptibility assay, using a fluorescence-based NA inhibition assay, was conducted on the 3 NA/H275Y viruses and 26 randomly selected isolates out of the 60 A(H1N1)pdm09 viruses collected during 2016 to 2019. The NA inhibition assay tested the susceptibility of these viruses to four types of NAIs: oseltamivir, peramivir, zanamivir, and laninamivir (Table 2). The 26 isolates without the NA/H275Y substitution in the NA segment demonstrated low IC_50_ values for peramivir, oseltamivir, zanamivir, and laninamivir, indicating sensitivity to all four drugs (Table 2). On the other hand, the influenza A(H1N1)pdm09 isolates with NA/H275Y showed elevated IC_50_ values for peramivir (5.60 nM, 5.29 nM, and 10.66 nM; 75.57–152.28-fold change) and oseltamivir (184.70 nM, 159.65 nM, and 263.48 nM; 301.22–497.13-fold change), but not for zanamivir (0.33 nM, 0.30 nM, and 0.49 nM, 0.79–1.2-fold change) and laninamivir (0.50 nM, 0.45 nM, and 0.64 nM, 1.51–2.13-fold change) (Table 2 and Table S8).

## 4. Discussion

This study represented the comprehensive whole-genome sequencing analysis of influenza A(H1N1)pdm09 viruses circulating in Myanmar during the 2015 to 2019 seasons. Phylogenetic trees indicated that viruses divided for distinct clades, 6B, 6B.1, 6B.1A, and 6B.1A.5a, from 2015 to 2019. We compared genetic differences between circulating strains and each season’s SH vaccine strains using whole-genome sequencing. The influenza A(H1N1)pdm09 viruses in Myanmar were found to have similar substitutions to those reported globally and mismatched with SH vaccine strains [31,32,33,34]. One virus, with an oseltamivir-resistant NA/H275Y substitution, was identified in 2017 and two were in 2019. They are highly suspected of being transmitted in the community. In addition, a virus with intra-subtype reassortment was detected in 2015 in our study.

The ladder-like phylogenetic tree of the HA gene in Myanmar present from 2015 to 2019 shows strains grouped into distinct clades: 6B, 6B.1, 6B.1A, and 6B.1A.5a. Each clade was defined by key amino acid substitutions. Viruses, collected in Myanmar during 2015, had D97N, K163Q, S185T, S203T, A256T, and K283E substitutions and belonged to the 6B clade. The National Institute of Infectious Diseases (NIID) in Japan registered seven strains collected from Myanmar between July and August 2015 without specifying the region. Our analysis showed that these strains fell into the 6B clade, the same clade as our strains (GISAID registration numbers: EPI_ISL_200637-39, 200642-45). Strains in 2016 obtained the S84N, S162N, and I216T substitutions and progressed to the 6B.1 clade, similar to the majority of viruses from South Asia, according to the nextflu report [35]. In 2017, viruses from Myanmar underwent evolution by acquiring the S74R, S164T, and I295V substitutions which characterize the 6B.1A clade, the same as with globally circulated strains [36]. Consequently, in 2019, viruses in Myanmar with the N129D and T185I substitutions evolved into the 6B.1A.5a clade [34]. The NIID also reported three strains collected in July and August 2019 in Myanmar, which fell into the same 6B.1A.5a clade (EPI_ISL_411916-8). Although the available information is not sufficient, these results indicate that similar strains circulated in Myanmar during the relevant year. Since the HA glycoprotein plays a key role in influenza virus antigenicity, influenza vaccines are targeting the HA. The accumulation of substitutions in the HA protein, particularly on the antigenic sites, receptor binding sites, and the surrounding region, has allowed the continuous evolution and the emergence of new influenza virus strains [37]. During our study, substitutions near the receptor binding site of the HA were observed: the S185T was observed among viruses circulating between 2015 and 2016, and the S183P in the 2019 strains. These substitutions have an important effect on the antigenic properties of influenza A(H1N1)pdm09. Additionally, the representative viruses of circulated clades during the 2016 season were poorly inhibited by some post-vaccination adult human serum; furthermore, the geometric mean post-vaccination HI titers of pediatric sera against some representative circulated A(H1N1)pdm09 clade viruses were significantly reduced compared to HI titers for the A/California/7/2009 virus vaccine.

As a result, A/California/7/2009, which was used as a vaccine strain until the 2016 season, was updated to A/Michigan/45/2015, starting from 2017 [38]. In 2019, the WHO reported that the majority of circulated viruses carried S74R, S164T, S183P, and I295V substitutions, as seen in A/Brisbane/02/2018 [39]. Similarly, HI assays with post-infection ferret antisera from circulating viruses were antigenically similar to the egg-propagated A/Brisbane/02/2018, which was recommended as a new vaccine strain for the 2020 influenza season [39]. These findings indicate that the SH vaccine strains in relevant years did not match with the circulating strains in Myanmar during the 2015–2019 seasons, although serological antigenic testing was not conducted in this study. Similar findings of influenza A(H1N1)pdm09 viruses were reported in China from 2014 to 2018 [40]. A report from India has shown that A(H1N1)pdm09 viruses circulating in India during 2017–2018 showed an amino acid identity of 98.74–99.28% with Michigan/45/2015, a 2017–2019 SH vaccine strain [41]. A study from Thailand revealed that A(H1N1)pdm09 Thai strains circulating between 2017 and 2020 had evolved away from their vaccine strain and suggested that the existing A(H1N1)pdm09 strains circulating in recent years have genetically drifted from their vaccine strains [37]. These findings underscore the importance of monitoring circulating virus strains and vaccine strains on a global scale.

The full-genome phylogenic tree of A(H1N1)pdm09 viruses from Myanmar in 2015–2019 demonstrated the incongruent pattern of the A/Yangon/15M43 virus among eight segments and therefore suggested the presence of minor reassortment during the 2015 season. The virus belonged to the 6B clade, in the HA, PB1, PA, NA, and MP segments, and became closer to the newer subclade 6B.1 in the PB2, NP, and NS segments. Similar findings from two A(H3N2) viruses from Myanmar in the 2016 season were reported by our group and in late 2016 or early 2017 by a group in the USA [17,42]. The intra-subtype reassortment of the former A(H1N1) was reported throughout 1918–2006, during which influenza A(H1N1) similarly evolved by changing the topological positions of its segments [43]. Moreover, some studies revealed that antigenically novel, highly transmissible, and drug resistant reassortant viruses occurred as the result of intra-subtype reassortments [43,44,45]. Full-genome sequencing of Myanmar strains is essential to understand reassortment and its role in influenza virus evolution.

A total of three viruses, one virus in 2017 and two viruses in 2019, with the H275Y substitution in NA that confer resistance to oseltamivir without a history of NAI administration, were detected in this study. These viruses exhibited high reduced inhibition (HRI) by oseltamivir (301.22–497.13 fold-increase in IC_50_) and reduced inhibition/high reduced inhibition (RI/HRI) by peramivir (75.57–152.28 fold-increase in IC_50_) without affecting the susceptibility to zanamivir and laninamivir. The first case of H275Y resistance to oseltamivir and peramivir without prior antiviral administration in Myanmar was reported in 2017 by our group [16]. In 2019, two additional NA/H275Y cases with unknown histories of NAI administration were detected, suspected to be transmitted in the community from those previously treated with oseltamivir. Since the initial global analysis of 2013–2014 data, similar findings of detecting a cluster of A(H1N1)pdm09 viruses with the NA H275Y substitution from patients with no previous exposure to antivirals had already been reported in other countries such as China, Japan, and the United States [46]. The sixth global update of data reported in 2020 showed 19 of the 48 H275Y cases had not received any NAIs prior to sample collection in the 2017–2018 period, suggesting human-to-human transmission of the H275Y variants [47]. These facts highlight the necessity for the continuous monitoring of the influenza virus and the close surveillance of antiviral drug resistance in Myanmar.

This study has some limitations. First, our study sites were limited to only two regions in Myanmar, which does not allow us to demonstrate findings at the country level. Second, in this study, no data were available regarding risk factors associated with disease severity because only outpatients were enrolled and we did not conduct a population-based study. Third, we implemented genetic analysis on isolated viruses; however, it would be preferable to perform this analysis using clinical samples to avoid substitutions arising from serial passage. Fourth, we did not conduct a phylogeographic analysis to observe the global and regional transmission routes of influenza A(H1N1)pdm09 in Myanmar. Previously, we reported that influenza A(H1N1)pdm09 present in Myanmar between the 2010 and 2015 seasons was most likely originating from Europe [10]. We also reported that influenza A(H1N1)pdm09 viruses that caused a big community outbreak in 2017 were probably sourced from South Asia [16]. However, we did not perform the phylogeographic analysis in this study. Fifth, only half of the viruses underwent NAI susceptibility assay, as NGS analysis showed that all viruses, except for the three with the NA/H275Y substitution, did not exhibit any known substitutions in the NA segment that confer resistance to NAIs. We concluded that conducting susceptibility assays on approximately 30 viruses was sufficient to calculate the median IC_50_ value and determine the fold change for the NA/H275Y viruses. Sixth, since influenza vaccination coverage in Myanmar is unclear, it has been impossible to assess its impact. It is important to examine the characteristic substitutions of viruses in Myanmar and the timing of clade introductions to understand which regions the influenza viruses in Myanmar are coming from and which regions they are impacting.

## Figures and Tables

**Table 1:** List of substitution differences in HA segment between Southern Hemisphere vaccine strains and Myanmar 2015–2019 viruses.

Strain	Clade	Amino Acid Substitutions in Each Nucleotide Position																
		35	61	**74**	**83**	**84**	**97**	**129**	143	146	155	**162**	**163**	**164**	**183**	**185**	**203**	**215**
A/California/7/2009 ^a^	1				**P**	**S**	**D**		S			**S**	**K**			**S**	**S**	**A**
2015 Myanmar viruses	6B				**S**	N	**N**		G				**Q**			**T**	**T**	
2016 Myanmar viruses	6B.1				**S**	**N**	**N**					**N**	**Q**			**T**	**T**	**G**
A/Michigan/45/2015 ^b^	6B.1	D	I	**S**				**N**		K	G			**S**	**S**	**T**		
2017 Myanmar viruses	6B.1A	G	V	**R**							E			**T**				
2019 Myanmar viruses	6B.1A.5a			**R**				**D**		R				**T**	**P**	**I**		
		**216**	**256**	261	**283**	**295**	**321**	370	**374**	391	449	**451**	454	478	**499**	501	528	**556**
A/California/7/2009 ^a^	1	**I**	**A**		**K**		**I**	N	**E**	T		**S**	K		**E**			**S**
2015 Myanmar viruses	6B		**T**		**E**		**V**	S	**K**	I		**N**	R		**K**			I
2016 Myanmar viruses	6B.1	**T**	**T**		**E**		**V**		**K**			**N**			**K**			**I**
A/Michigan/45/2015 ^b^	6B.1			A		**I**					V			S		D	V	
2017 Myanmar viruses	6B.1A			V		**V**					I							
2019 Myanmar viruses	6B.1A.5a					**V**								N		Y	I	

^a^ Vaccine strain for 2015 and 2016, Southern Hemisphere. ^b^ Vaccine strain for 2017 and 2019, Southern Hemisphere. Amino acid substitutions in bold represent common substitutions for all isolates in each season, whereas those in normal font represent additional sporadic substitutions observed in some of the relevant season strains compared to the vaccine strain for the season.

**Table 2:** Antiviral susceptibility characteristics of influenza A(H1N1)pdm09 viruses, collected in Myanmar between 2016 and 2019, to four NAIs.

	IC_50_ (nM) Median, Min–Max [Fold Change] *			
NAIs	Peramivir	Oseltamivir	Zanamivir	Laninamivir
Viruses without NA/H275Y substitution, N = 26	0.07, 0.06–0.18 [0.85–2.59]	0.53, 0.42–4.50 [0.80–8.49]	0.38, 0.31–3.71 [0.82–9.75]	0.30, 0.21–0.80 [0.70–2.67]
Viruses possessing NA/H275Y substitution, N = 3	5.60, 5.29–10.66 [75.57–152.28]	184.70, 159.65–263.48 [301.22–497.13]	0.33, 0.30–0.49 [0.79–1.29]	0.50, 0.45–0.64 [1.51–2.13]

* The fold change was compared to a median IC_50_ of viruses of the same type/subtype without NA/H275Y collected during our study. The minimum and maximum fold changes are shown.

## Data Availability

The whole-genome nucleotide sequences generated in this study have been deposited in the Global Initiative on Sharing All Influenza Data (GISAID) EpiFlu database with accession numbers EPI_ISL_12870031–EPI_ISL_12995850.

## Supplementary Materials

The following supporting information can be downloaded at https://www.mdpi.com/article/10.3390/v16081300/s1, Supplementary Figure S1. The phylogenetic relationships of the HA segment in 210 A(H1N1)pdm09 viruses from Myanmar (2015–2019) and the closest BLAST hits from different regions worldwide. The maximum-likelihood tree was inferred from these HA gene sequences and compared to the Southern Hemisphere vaccine strains with clade classifications recommended by the World Health Organization (shown in bold). Strains from different regions are indicated in various colors. Bootstrap values > 70% are displayed at the nodes. Supplementary Table S1. List of substitution differences in NA segment between Southern Hemisphere vaccine strains and Myanmar 2015–2019 viruses. Supplementary Table S2. List of substitution differences in PB2 segment between Southern Hemisphere vaccine strains and Myanmar 2015–2019 viruses. Supplementary Table S3. List of substitution differences in PB1 segment between Southern Hemisphere vaccine strains and Myanmar 2015–2019 viruses. Supplementary Table S4. List of substitution differences in PA segment between Southern Hemisphere vaccine strains and Myanmar 2015–2019 viruses. Supplementary Table S5. List of substitution differences in NP segment between Southern Hemisphere vaccine strains and Myanmar 2015–2019 viruses. Supplementary Table S6. List of substitution differences in MP segment between Southern Hemisphere vaccine strains and Myanmar 2015–2019 viruses. Supplementary Table S7. List of substitution differences in NS segment between Southern Hemisphere vaccine strains and Myanmar 2015–2019 viruses. Supplementary Table S8. Antiviral susceptibility of influenza A(H1N1)pdm09 isolates to four NAIs, determined using the NA inhibition assay, for 29 viruses collected in Myanmar between 2016 and 2019.
