# Supplementary material for: Whole-Genome Analysis of the Influenza A(H1N1)pdm09 Viruses Isolated from Influenza-like Illness Outpatients in Myanmar and Community-Acquired Oseltamivir-Resistant Strains Present from 2015 to 2019
Source: Viruses. 2024 Aug 15;16(8):1300. doi: 10.3390/v16081300 (PMC11360699; doi:10.3390/v16081300)
Supplement: Supplementary file 1 [file viruses-16-01300-s001.zip › Supplementary Table S1 to S7.pdf]

Supplementary Table S1. List of substitution differences in NA segment between Southern Hemisphere vaccine strains and Myanmar 2015-2019 viruses.

**Representative strain**	**Clade**	**Amino Acid Mutations in Each Nucleotide Position**																		
		8	**13**	16	**34**	**40**	**44**	**51**	**74**	**77**	**81**	**117**	**173**	**188**	**200**	203	214	**241**	**248**	**264**
A/California/7/2009[a]	1	I	**V**	T	**I**	**L**	**N**					**I**			**N**			**V**	**N**	**V**
2015 Myanmar viruses	6B	T		I	**V**	**I**	**S**					**M**			**S**			**I**	**D**	
2016 Myanmar viruses	6B.1		**I**		**V**	**I**	**S**								**S**			**I**	**D**	**I**
A/Michigan/45/2015[b]	6B.1				V			**Q**	**F**	**G**	V		**R**	**I**		V	D			
2017 Myanmar viruses	6B.1A									**R**	A		**K**	**T**						
2019 Myanmar viruses	6B.1A.5a				I			**K**	**S**	**R**	**A**			**T**		M	G			

**Representative strain**	**Clade**	**Amino Acid Mutations in Each Nucleotide Position**																		
		267	**270**	275	**314**	**321**	**341**	354	**365**	**369**	**386**	**389**	397	**416**	**432**	434	**449**	**451**	**452**	453
A/California/7/2009[a]	1	V	N		**I**	**I**	**N**	G	**I**	**N**	**N**		N		**K**			**D**		
2015 Myanmar viruses	6B	I				**V**	**S**	D	**T**	**K**	**K**				**E**					
2016 Myanmar viruses	6B.1		**K**		**M**	**V**				**K**	**K**		S		**E**			**G**		
A/Michigan/45/2015[b]	6B.1			H								**I**		**D**		N	**N**		**T**	V
2017 Myanmar viruses	6B.1A																**D**			
2019 Myanmar viruses	6B.1A.5a			Y								**K**		**N**		K	**D**		**I**	M

[a] Vaccine strain for 2015 and 2016 and [b] Vaccine strain for 2017 and 2019.

Amino acid substitutions in bold represent common mutations for all isolates in each season, whereas those in normal font represent additional sporadic mutations observed in some of the relevant season strains.

Supplementary Table S2. List of substitution differences in PB2 segment between Southern Hemisphere vaccine strains and Myanmar 2015-2019 viruses.

**Representative strain**	**Clade**	**Amino Acid Mutations in Each Nucleotide Position**														
		**59**	69	**71**	77	86	96	**107**	112	161	**189**	**200**	**230**	248	**298**	**304**
A/California/7/2009[a]	1	**R**		**M**		T		**N**	S	A	**A**	**D**			**R**	**R**
2015 Myanmar viruses	6B	**K**		**I**		I		**S**	N	T	**T**	**N**			**K**	K
2016 Myanmar viruses	6B.1	**K**		**I**								**N**			**K**	**K**
A/Michigan/45/2015[b]	6B.1		M	**I**	E	**T**	V						**G**	M		**R**
2017 Myanmar viruses	6B.1A			**T**			I									**K**
2019 Myanmar viruses	6B.1A.5a		V		**G**	**I**							**S**	V		**K**

**Representative strain**	**Clade**	**Amino Acid Mutations in Each Nucleotide Position**														
		313	**349**	**359**	373	**403**	418	**458**	478	480	516	572	648	**664**	**672**	**736**
A/California/7/2009[a]	1		**V**	**I**	R			**S**	M							**V**
2015 Myanmar viruses	6B		**M**	**L**	K			T	I							**I**
2016 Myanmar viruses	6B.1		**M**	**L**				**T**								**I**
A/Michigan/45/2015[b]	6B.1	V				**T**	A	**P**		L	V	D	S	**I**	**V**	
2017 Myanmar viruses	6B.1A	I				**I**	T	**I**		M	I	E		**N**		
2019 Myanmar viruses	6B.1A.5a					**I**		**I**			I		T	**N**	**I**	

[a] Vaccine strain for 2015 and 2016 and [b] Vaccine strain for 2017 and 2019.

Amino acid substitutions in bold represent common mutations for all isolates in each season, whereas those in normal font represent additional sporadic mutations observed in some of the relevant season strains

Supplementary Table S3. List of substitution differences in PB1 segment between Southern Hemisphere vaccine strains and Myanmar 2015-2019 viruses.

**Representative strain**	**Clade**	**Amino Acid Mutations in Each Nucleotide Position**																				
		**12**	14	52	113	**154**	**175**	179	190	**200**	**211**	212	245	248	298	322	327	328	339	364	**375**	**386**
A/California/7/2009[a]	1	I		K	V	**G**	**N**	I		V	**R**	L	W	D	I	I	R		M	I		K
2015 Myanmar viruses	6B	V		R	A	**D**	**D**	M		I	**K**	V	R	N	L	V	K		I	L		R
2016 Myanmar viruses	6B.1					**D**	**D**															
A/Michigan/45/2015[b]	6B.1	**I**	A						R	**V**								N			**S**	**K**
2017 Myanmar viruses	6B.1A	**V**																D			**N**	
2019 Myanmar viruses	6B.1A.5a		V						I	**I**												**R**

**Representative strain**	**Clade**	**Amino Acid Mutations in Each Nucleotide Position**																				
		393	**397**	**435**	480	508	517	537	576	581	586	587	618	619	628	638	643	688	709	728	753	**761**
A/California/7/2009[a]	1	R	**I**	**I**		E	V		L	D	K	V	D	D		D	A	M	V	V	L	**N**
2015 Myanmar viruses	6B		**M**	**T**		D	I		I	G	R	T	E	N		E			I	I	F	**D**
2016 Myanmar viruses	6B.1	K	**M**	**T**													T	I				
A/Michigan/45/2015[b]	6B.1				K			N							L							
2017 Myanmar viruses	6B.1A				R			S														
2019 Myanmar viruses	6B.1A.5a														M							

[a] Vaccine strain for 2015 and 2016 and [b] Vaccine strain for 2017 and 2019.

Amino acid substitutions in bold represent common mutations for all isolates in each season, whereas those in normal font represent additional sporadic mutations observed in some of the relevant season strains.

Supplementary Table S4. List of substitution differences in PA segment between Southern Hemisphere vaccine strains and Myanmar 2015-2019 viruses.

**Representative strain**	**Clade**	**Amino Acid Mutations in Each Nucleotide Position**																		
		55	63	**100**	142	216	**224**	**225**	256	277	**321**	322	**330**	**354**	**361**	**362**	364	452	**505**	**531**
A/California/7/2009[a]	1	D	V	**V**	K		**P**		K	H	**N**	I	**I**	I	**K**	**R**		H		**R**
2015 Myanmar viruses	6B	N	A	**I**			**S**		Q	L	**K**	V	**V**	V	**R**	**K**		Q		**K**
2016 Myanmar viruses	6B.1			**I**	E		**S**				**K**		**V**			**K**				
A/Michigan/45/2015[b]	6B.1					D		**S**						**I**			S		**I**	
2017 Myanmar viruses	6B.1A					N											N			
2019 Myanmar viruses	6B.1A.5a							**C**						**V**					**V**	

**Representative strain**	**Clade**	**Amino Acid Mutations in Each Nucleotide Position**									
		561	614	615	616	617	**649**	650	**682**	691	719
A/California/7/2009[a]	1	M			S	E	**L**		**D**		W
2015 Myanmar viruses	6B	L				A	**I**		**N**		
2016 Myanmar viruses	6B.1				L						R
A/Michigan/45/2015[b]	6B.1		N	K				Y		E	
2017 Myanmar viruses	6B.1A							C		G	
2019 Myanmar viruses	6B.1A.5a		K	R							

[a] Vaccine strain for 2015 and 2016 and [b] Vaccine strain for 2017 and 2019.

Amino acid substitutions in bold represent common mutations for all isolates in each season, whereas those in normal font represent additional sporadic mutations observed in some of the relevant season strains.

Supplementary Table S5. List of substitution differences in NP segment between Southern Hemisphere vaccine strains and Myanmar 2015-2019 viruses.

**Representative strain**	**Clade**	**Amino Acid Mutations in Each Nucleotide Position**																		
		11	**33**	42	45	63	64	**111**	**112**	116	120	128	130	139	142	145	228	228	250	291
A/California/7/2009[a]	1	A	**A**	R	G	Y	D	**V**	**G**	M	I	R	V		A		V	V	M	V
2015 Myanmar viruses	6B		T	K	D	H	E	**I**	**D**	T	V		I		S		I	G	V	A
2016 Myanmar viruses	6B.1	V	**T**					**I**	**D**			K								
A/Michigan/45/2015[b]	6B.1													D		T				
2017 Myanmar viruses	6B.1A																			
2019 Myanmar viruses	6B.1A.5a													E		A				

**Representative strain**	**Clade**	**Amino Acid Mutations in Each Nucleotide Position**																
		294	354	355	361	364	382	383	384	386	388	395	411	**436**	**455**	470	**509**	**511**
A/California/7/2009[a]	1	L	V	S	K	I	V	E	T	D	N	R	K	**V**	**V**	Q	**S**	**G**
2015 Myanmar viruses	6B	P	L	L	T	S	M	D	N	G	S	G	R	**I**	**I**	R	**N**	**R**
2016 Myanmar viruses	6B.1																**N**	**R**
A/Michigan/45/2015[b]	6B.1													**V**				
2017 Myanmar viruses	6B.1A																	
2019 Myanmar viruses	6B.1A.5a													**I**				

[a] Vaccine strain for 2015 and 2016 and [b] Vaccine strain for 2017 and 2019.

Amino acid substitutions in bold represent common mutations for all isolates in each season, whereas those in normal font represent additional sporadic mutations observed in some of the relevant season strains.

Supplementary Table S6. List of substitution differences in MP segment between Southern Hemisphere vaccine strains and Myanmar 2015-2019 viruses.

Representative strain	Clade	Amino Acid Mutations in Each Nucleotide Position																		
		15	30	30	**80**	95	101	115	116	121	133	**167**	**192**	205	207	**208**	209	214	218	227
A/California/7/2009[a]	1	I	S	S	**V**	R	K	V	S	T	N	**T**	**M**	V	N	**Q**	T	H	T	A
2015 Myanmar viruses	6B	V	D	N	**I**	K	R	I	A	A	S	**A**	**V**	I	S	K	A	Q	A	T
2016 Myanmar viruses	6B.1				**I**								**V**			**K**				
A/Michigan/45/2015[b]	6B.1																			
2017 Myanmar viruses	6B.1A																			
2019 Myanmar viruses	6B.1A.5a																			

Representative strain	Clade	Amino Acid Mutations in Each Nucleotide Position																		
		**230**	239	253	254	255	256	257	258	259	**260**	**262**	266	271	**278**	281	283	285	289	295
A/California/7/2009[a]	1	**K**	A	S	S	R	H		S	K	**Y**		P	Y	**Y**	S	I	F	A	A
2015 Myanmar viruses	6B	**R**	T	P	A	C	C		R	E	**H**		L	S	H	T	T	P	P	T
2016 Myanmar viruses	6B.1	**R**									**H**				**H**					
A/Michigan/45/2015[b]	6B.1							C				**W**								
2017 Myanmar viruses	6B.1A							Y				**R**								
2019 Myanmar viruses	6B.1A.5a																			

Representative strain	Clade	Amino Acid Mutations in Each Nucleotide Position					
		297	303	**305**	309	312	313
A/California/7/2009[a]	1	H	T	**T**	C	R	W
2015 Myanmar viruses	6B	Y	K	**A**	R	Q	Q
2016 Myanmar viruses	6B.1			**A**			
A/Michigan/45/2015[b]	6B.1						
2017 Myanmar viruses	6B.1A						
2019 Myanmar viruses	6B.1A.5a						

[a] Vaccine strain for 2015 and 2016 and [b] Vaccine strain for 2017 and 2019.
Amino acid substitutions in bold represent common mutations for all isolates in each season, whereas those in normal font represent additional sporadic mutations observed in some of the relevant season strains.

Supplementary Table S7. List of substitution differences in NS segment between Southern Hemisphere vaccine strains and Myanmar 2015-2019 viruses.

**Representative strain**	**Clade**	**Amino Acid Mutations in Each Nucleotide Position**																		
		**2**	9	20	**55**	**65**	**80**	**90**	92	**123**	**125**	**131**	**155**	164	**205**	**220**	**223**	**224**	225	**233**
A/California/7/2009[a]	1	**D**	F	K	**E**			**L**		**I**	**E**	**K**			**N**	**K**	**R**	**A**	I	
2015 Myanmar viruses	6B		L	R	**K**			**I**		**V**		**E**			**S**	**E**	G	**T**	T	
2016 Myanmar viruses	6B.1	**E**			**K**			**I**		**V**	**D**	**E**			**S**	**E**	**G**	**T**		
A/Michigan/45/2015[b]	6B.1					**M**	**T**		D				**A**	P		**E**				**V**
2017 Myanmar viruses	6B.1A					**V**								H						
2019 Myanmar viruses	6B.1A.5a					**V**	**A**		G				**T**			**K**				**M**

**Representative strain**	**Clade**	**Amino Acid Mutations in Each Nucleotide Position**					
		**238**	**239**	**244**	257	**263**	270
A/California/7/2009[a]	1	**A**		**D**		**T**	V
2015 Myanmar viruses	6B	T				A	I
2016 Myanmar viruses	6B.1	**T**		**N**		**A**	
A/Michigan/45/2015[b]	6B.1		**A**		T		
2017 Myanmar viruses	6B.1A		**T**				
2019 Myanmar viruses	6B.1A.5a		**T**		A		

[a] Vaccine strain for 2015 and 2016 and [b] Vaccine strain for 2017 and 2019.
Amino acid substitutions in bold represent common mutations for all isolates in each season, whereas those in normal font represent additional sporadic mutations observed in some of the relevant season strains.
