# Supplementary material for: Whole-Genome Analysis of the Influenza A(H1N1)pdm09 Viruses Isolated from Influenza-like Illness Outpatients in Myanmar and Community-Acquired Oseltamivir-Resistant Strains Present from 2015 to 2019
Source: Viruses. 2024 Aug 15;16(8):1300. doi: 10.3390/v16081300 (PMC11360699; doi:10.3390/v16081300)
Supplement: Supplementary file 1 [file viruses-16-01300-s001.zip › Supplementary Table S8.pdf]

Supplementary Table S8. Antiviral susceptibility of influenza A(H1N1)pdm09 isolates to four NAIs, determined using the NA inhibition assay, for 29 viruses collected in Myanmar between 2016 and 2019.

No.	Strain name	IC_{50} values for each NAIs [nM]			
		Peramivir	Oseltamivir	Zanamivir	Laninamivir
**1**	A/Pyinmana/16M172	0.09	0.89	0.45	0.32
**2**	A/Yangon/17M001	0.06	0.43	0.35	0.25
**3**	A/Yangon/17M004	0.06	0.46	0.36	0.25
**4**	A/Yangon/17M010	0.06	0.44	0.35	0.27
**5**	A/Yangon/17M016	0.07	0.45	0.36	0.26
**6**	A/Yangon/17M033	0.06	0.46	0.34	0.21
**7**	A/Yangon/17M044	0.06	0.47	0.37	0.28
**8**	A/Yangon/17M063	0.06	0.42	0.37	0.26
**9**	A/Yangon/17M082	0.07	0.49	0.36	0.28
**10**	A/Yangon/17M093	0.07	0.45	0.33	0.30
**11**	A/Yangon/17M108	0.06	0.46	0.38	0.26
**12**	A/Yangon/17M141	0.07	0.57	0.38	0.26
**13**	A/Yangon/17M158	0.07	0.52	0.40	0.30
**14**	A/Pyinmana/17M219	0.07	0.49	0.40	0.28
**15**	A/Pyinmana /17M307/2017	10.66	184.70	0.49	0.50
**16**	A/Yangon/19M001/2019	0.07	0.58	0.35	0.35
**17**	A/Yangon/19M007/2019	0.08	0.92	0.66	0.32
**18**	A/Yangon/19M018/2019	0.08	0.54	0.31	0.26
**19**	A/Yangon/19M027/2019	5.29	159.65	0.33	0.45
**20**	A/Yangon/19M044/2019	0.08	0.62	0.58	0.36
**21**	A/Yangon/19M052/2019	5.60	263.48	0.30	0.64
**22**	A/Yangon/19M113/2019	0.08	0.52	0.38	0.32
**23**	A/Yangon/19M152/2019	0.15	2.11	1.10	0.47
**24**	A/Yangon/19M236/2019	0.18	4.50	3.59	0.80
**25**	A/Yangon/19M240/2019	0.15	3.62	0.79	0.72
**26**	A/Yangon/19M242/2019	0.10	1.06	0.73	0.41
**27**	A/Yangon/19M253/2019	0.17	4.15	3.71	0.77
**28**	A/Yangon/19M292/2019	0.10	0.72	0.72	0.61
**29**	A/Yangon/19M294/2019	0.15	1.29	0.87	0.72

Abbreviations: NAIs neuraminidase inhibitors; IC_{50} half maximal inhibitory concentration.
