# Supplementary material for: Whole-Genome Analysis of the Influenza A(H1N1)pdm09 Viruses Isolated from Influenza-like Illness Outpatients in Myanmar and Community-Acquired Oseltamivir-Resistant Strains Present from 2015 to 2019
Source: Viruses. 2024 Aug 15;16(8):1300. doi: 10.3390/v16081300 (PMC11360699; doi:10.3390/v16081300)

Supplementary Figure S1. The phylogenetic relationships of the HA segment in 210 A(H1N1)pdm09 viruses from Myanmar (2015–2019) and the closest BLAST hits from different regions worldwide. The maximum-likelihood tree was inferred from these HA gene sequences and compared to the Southern Hemisphere vaccine strains with clade classifications recommended by the World Health Organization (shown in bold). Strains from different regions are indicated in various colors. Bootstrap values > 70% are displayed at the nodes.

HA segment of A(H1N1)pdm09

Reference strains
Vaccine strains
Myanmar
USA
Southeast Asia
China
South America
Europe
South Asia
Oceania
Africa

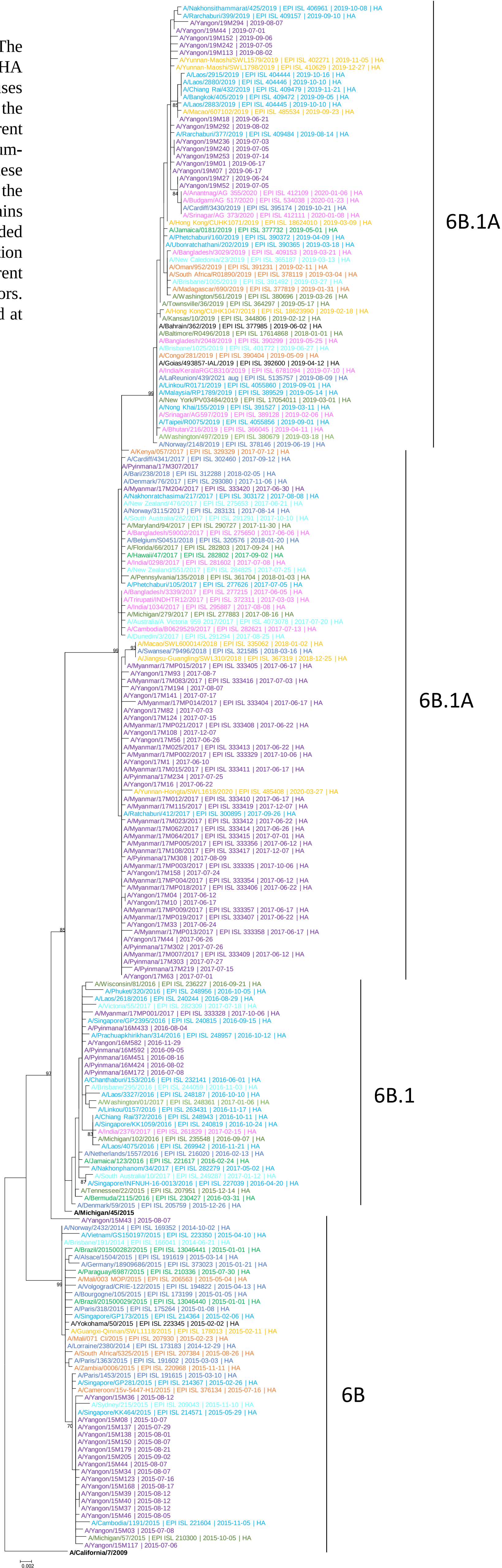

Supplement: Supplementary file 1 [file Supplementary Figure S1.pdf › Supplementary Figure S1.pdf]
